# Case Report: A collateral-supplying major septal artery occlusion: electromechanical consequences leading to low left ventricular ejection fraction and late-onset complete atrioventricular block

**DOI:** 10.3389/fcvm.2026.1760781

**Published:** 2026-02-13

**Authors:** Ali Hakan Konuş

**Affiliations:** Department of Cardiology, Bingöl State Hospital, Bingöl, Türkiye

**Keywords:** cardiac resynchronization therapy, complete atrioventricular block, heart failure, percutaneous coronary intervention, septal infarction

## Abstract

**Background:**

Septal arteries (SAs) are often considered minor branches, yet certain anatomies—particularly when a large septal artery (SA) provides collateral perfusion to a chronically occluded coronary vessel—carry substantial electromechanical risk. Abrupt loss of such a large SA during percutaneous coronary intervention (PCI) may compromise both conduction system perfusion and myocardial territories in selected anatomical settings. Although SA occlusion is typically benign, delayed complete atrioventricular (AV) block and severe left ventricular dysfunction are rarely reported complications.

**Case:**

A 62-year-old man presented with typical chest pain and was diagnosed with non–ST-elevation myocardial infarction. The baseline electrocardiogram showed normal sinus rhythm without conduction abnormalities. Coronary angiography revealed culprit proximal-mid left anterior descending artery (LAD) lesions and a chronic total occlusion of the right coronary artery, supplied by a large collateral-providing first SA. During intravascular ultrasound-guided PCI of the LAD, this major SA became unintentionally occluded despite protection with a jailed wire. Immediately afterward, serial electrocardiograms demonstrated a new bifascicular block pattern—complete right bundle branch block with right axis deviation—which persisted over the next two days. At 62 hours post-PCI, the patient developed late-onset complete AV block. Echocardiography showed a marked decline in left ventricular ejection fraction (LVEF) from 61% to 32%, consistent with new septal akinesia and inferior/inferolateral wall-motion abnormalities, suggesting a clinically significant ischemic insult following SA occlusion. Given the persistence of complete AV block requiring pacing support and the severely reduced LVEF, early cardiac resynchronization therapy with defibrillator (CRT-D) implantation was performed. The patient stabilized thereafter and was discharged in good condition.

**Conclusion:**

This case highlights that occlusion of a collateral-supplying major SA may result not only in conduction disturbances but also in significant left ventricular systolic dysfunction, producing an uncommon combined electromechanical presentation. Recognition of this high-risk anatomy during PCI planning—including an understanding that even anatomically appropriate protection strategies may not fully prevent septal branch loss—underscores the need for individualized, anatomy-guided side-branch protection, vigilant rhythm monitoring, and timely CRT-D implantation in selected patients.

## Introduction

Septal arteries (SAs) are traditionally regarded as small, non-major branches of the coronary circulation, and intentional septal artery (SA) occlusion is even used therapeutically in hypertrophic obstructive cardiomyopathy (1). Although SA occlusion—whether involving a septal branch or perforator—can affect the cardiac conduction system, given their role in supplying the His–Purkinje network, these events are generally described as hemodynamically well tolerated and rarely associated with clinically significant myocardial injury.

However, this benign perception does not apply to all anatomical settings. When an SA supplies critical downstream structures, the clinical consequence of its loss may be substantially greater than traditionally expected. In particular, sudden occlusion of a major SA supplying collateral flow may compromise the perfusion of both the conduction system and a large myocardial region simultaneously. In this context, late-onset complete atrioventricular (AV) block accompanied by a marked decline in left ventricular ejection fraction (LVEF) represents an uncommon and clinically important complication.

Here we describe a rare anatomical–clinical presentation of SA occlusion during left anterior descending artery (LAD) percutaneous coronary intervention (PCI), in which a collateral-supplying major septal branch sustaining a chronically occluded right coronary artery (RCA) was compromised and was followed by delayed complete AV block and marked left ventricular systolic dysfunction. In contrast to most previously reported septal branch occlusions, this case illustrates an electromechanical presentation extending beyond isolated conduction disturbances.

## Case report

**Figure 1:**
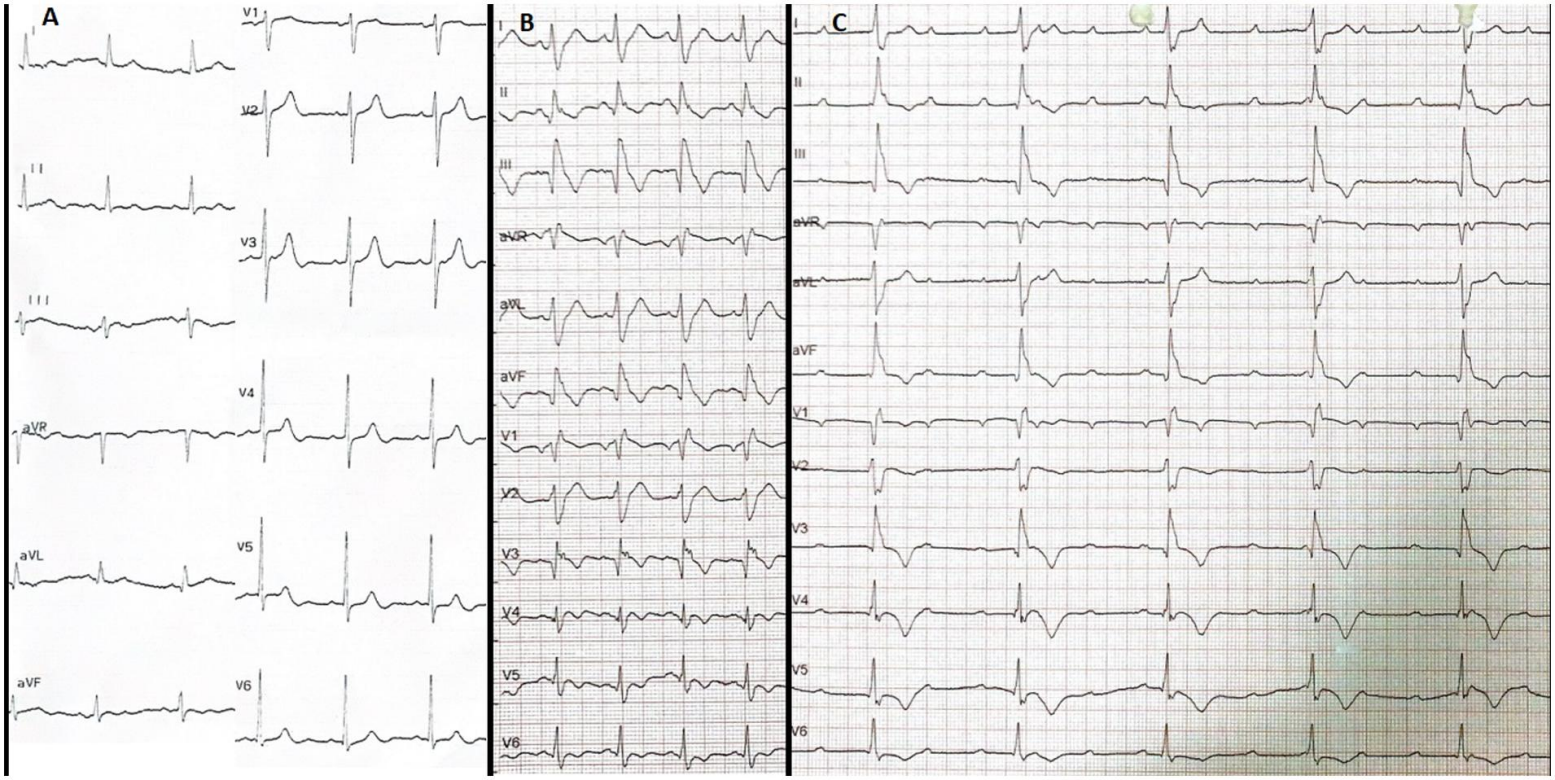
All ECGs of the case before CRT implantation. **(A)** Baseline normal ECG. **(B)** Early post-procedure ECG showing newly developed CRBBB with QRS duration of 168 ms, T wave negativity in the precordial (except V2) and inferior leads, pathological Q waves in the inferior leads, and Qr morphology in V1 and V3. **(C)** ECG on the third post-infarction day demonstrating complete AV block with persistent CRBBB and rightward QRS axis, confirming a bifascicular block pattern (CRBBB + features suggestive of LPFB) complicated by complete AV block, indicative of advanced conduction system injury following myocardial infarction. All electrocardiograms were recorded using standard calibration (10 mm/mV amplitude and 25 mm/s paper speed). ECG, electrocardiogram; CRBBB, complete right bundle branch block; LPFB, left posterior fascicular block; AV, atrioventricular; CRT, cardiac resynchronization therapy.

A 63-year-old man presented to the emergency department with typical chest pain. His medical history included hypertension and type 2 diabetes mellitus, without smoking or family history of coronary artery disease. The initial electrocardiogram (ECG) showed sinus rhythm without ischemic changes (Figure 1A). High-sensitivity cardiac troponin I (hs-cTnI) was elevated at 379 pg/mL (reference: <16.8 pg/mL). Transthoracic echocardiography (TTE) revealed a LVEF of 61%, concentric left ventricular hypertrophy, and no regional wall motion abnormalities. The patient was diagnosed with non–ST-segment elevation myocardial infarction (NSTEMI) and received guideline-directed therapy, including aspirin, ticagrelor, and intravenous unfractionated heparin.

**Figure 2:**
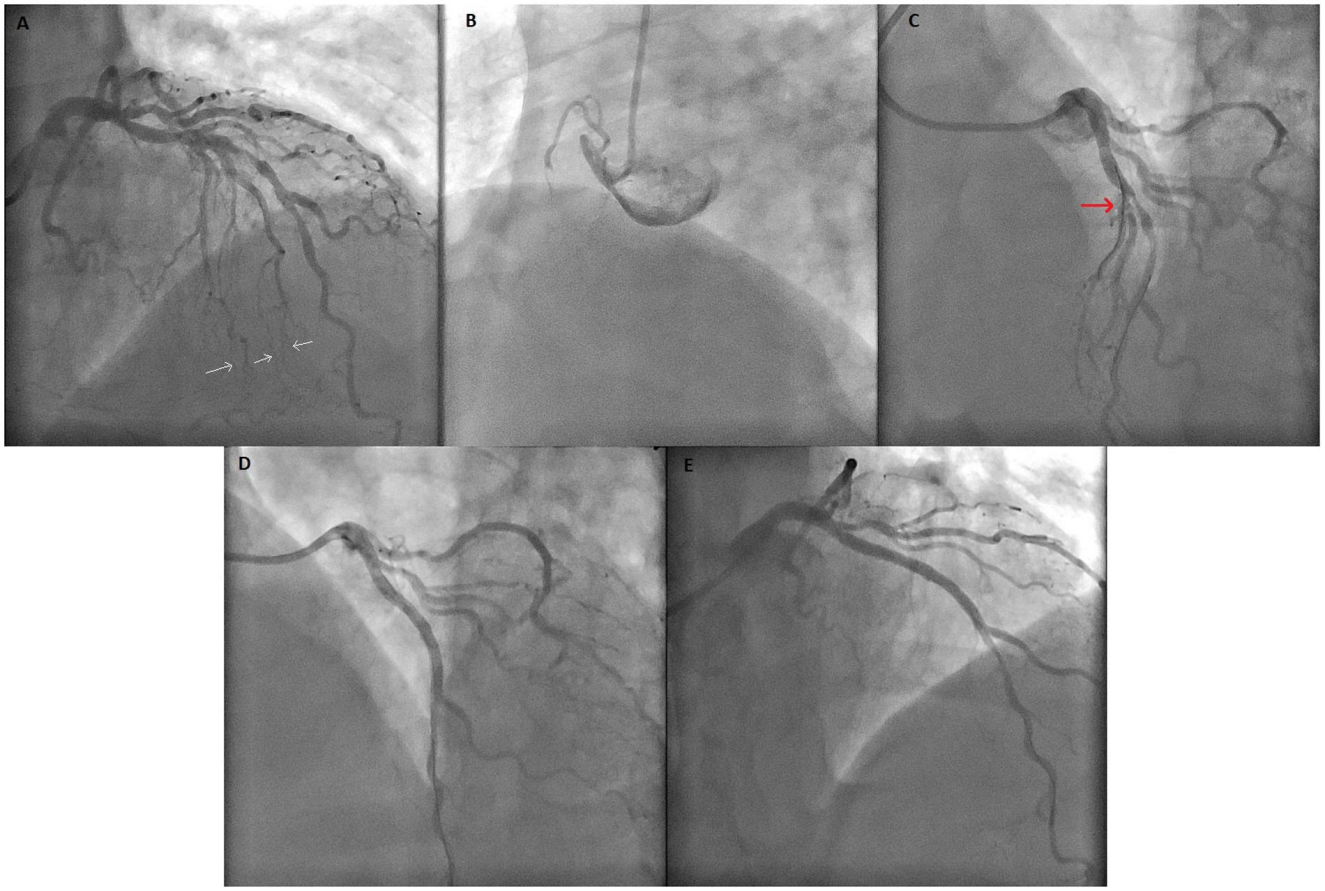
Coronary angiogram images of the case. **(A)** Coronary angiogram showing lesions of the LAD and a major SA containing extensive collateral vessels to the RCA. White arrows highlight the septal-to-RCA collateral vessels. **(B)** Image of CTO lesion of RCA. **(C)** Before PCI to LAD, major SA (red arrow) was protected with wire. **(D)** The coronary angiogram showing SA occlusion after PCI to the LAD. **(E)** Final image showing TIMI-0 flow in SA after unsuccessful wiring despite all attempts. PCI, percutaneous coronary intervention; LAD, left anterior descending artery; SA, septal artery; CTO, chronic total occlusion; TIMI, Thrombolysis In Myocardial Infarction; RCA, right coronary artery.

Coronary angiography revealed two severe consecutive lesions in the proximal–mid LAD and a chronic total occlusion (CTO) of the proximal RCA. The RCA territory was perfused by Rentrop grade 3 collateral flow from a large first SA arising from the LAD (Figure 2; Supplementary Video S1). Considering the coronary anatomy and patient preference, it was decided that PCI of the culprit LAD lesions and subsequent elective evaluation of the CTO in the proximal RCA would be the ideal approach.

Pre-PCI intravascular ultrasound (IVUS) evaluation demonstrated a narrow LAD–septal bifurcation angle and relatively low plaque burden at the septal ostium; therefore, a provisional strategy with septal protection was planned. Given these anatomical features, the jailed-wire technique was selected as the preferred protection method. A jailed-balloon technique was not chosen because the intramyocardial course and narrow takeoff of the septal branch raised concerns regarding incomplete balloon expansion and potential device entrapment. A 0.014-inch conventional guidewire was positioned in the SA for protection throughout the procedure. IVUS-guided PCI was then performed, and a 3.0 × 38 mm drug-eluting stent (Resolute Integrity) was implanted at 13 atm. After stent deployment, complete LAD patency was achieved; however, the SA was found to be totally occluded at its ostium despite the presence of the jailed wire (Figure 2).

The patient developed chest pain, and catheter movement caused by arm motion led to guiding catheter dislocation. After the catheter was repositioned, proximal optimization (POT) was performed with a 3.5 × 12 mm non-compliant balloon. IVUS confirmed adequate stent expansion and apposition. Despite administration of intracoronary nitroglycerin (100 μg), the SA remained occluded. Multiple guidewires (BMW, Runthrough, and Fielder XT) were sequentially used with the support of a FineDuo double-lumen microcatheter, but recanalization attempts were unsuccessful (Figure 2; Supplementary Video S2). IVUS demonstrated complete ostial occlusion due to plaque and/or carina shift, with no evidence of thrombus or dissection.

Following the 3 h procedure, the patient was transferred to the coronary care unit. His blood pressure was 95/62 mmHg and improved with intravenous fluids. The post-PCI ECG (Figure 1B) showed new-onset complete right bundle branch block (CRBBB) with a QRS duration of 168 ms, right axis deviation (isoelectric QRS in lead I, positive in aVF), T-wave inversion in the inferior and precordial leads (except V2), pathological Q waves in the inferior leads, and a Qr morphology in V1 and V3.

TTE demonstrated akinesia of the basal and mid septum, severe hypokinesia of the inferior and inferolateral walls, and an LVEF of 32%. Over the next 48 h, chest pain gradually subsided, and he remained hemodynamically stable. Peak hs-cTnI reached 6,451 pg/mL.

At 62 h post-PCI, complete AV block with a ventricular rate of 44 bpm developed (Figure 1C). The patient became hypotensive and vomited; therefore, a temporary transvenous pacemaker was immediately implanted via the right femoral vein, resulting in stabilization of hemodynamics. The complete AV block persisted whenever temporary pacing was withheld, and no reversible metabolic contributors were identified, as electrolytes, renal function, and hemoglobin remained within normal limits.

**Figure 3:**
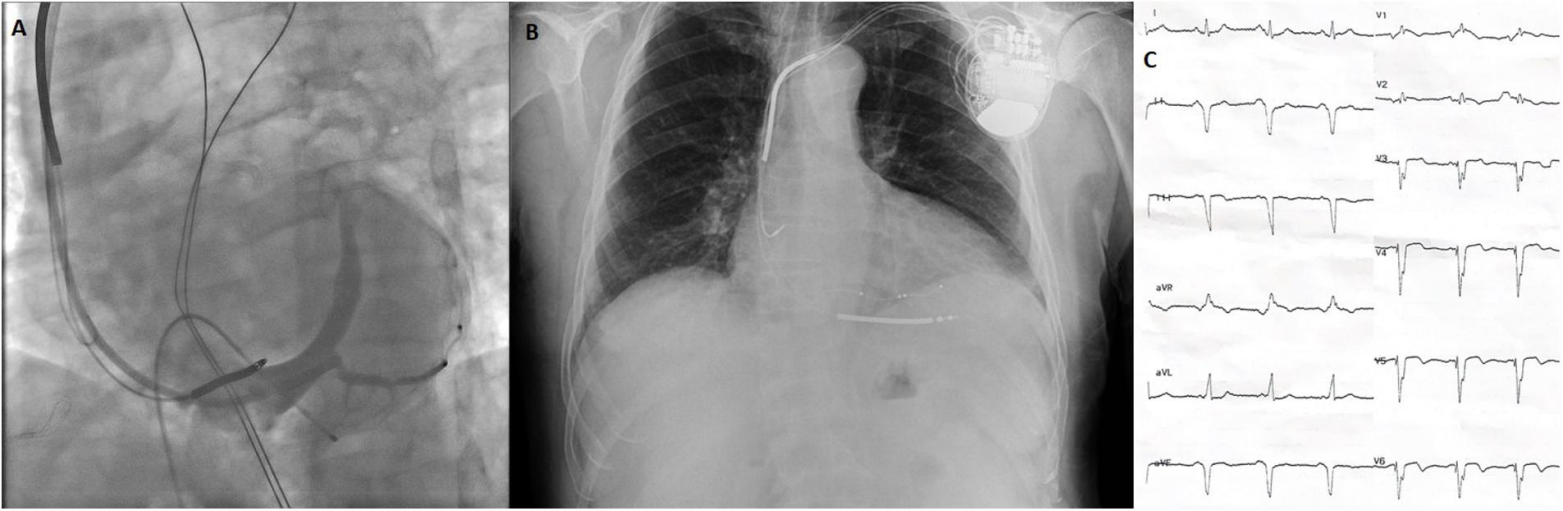
Images of battery and leads during and after CRT implantation and post-CRT ECG. **(A)** Coronary sinus angiogram under temporary transvenous pacemaker. **(B)** Postero-anterior chest x-ray after CRT implantation. **(C)** ECG after CRT implantation showing a biventricular paced rhythm with a QRS width 128 ms. *All electrocardiograms were recorded using standard calibration (10 mm/mV amplitude and 25 mm/s paper speed). ECG, electrocardiogram; CRT, cardiac resynchronization therapy.

**Figure 4:**
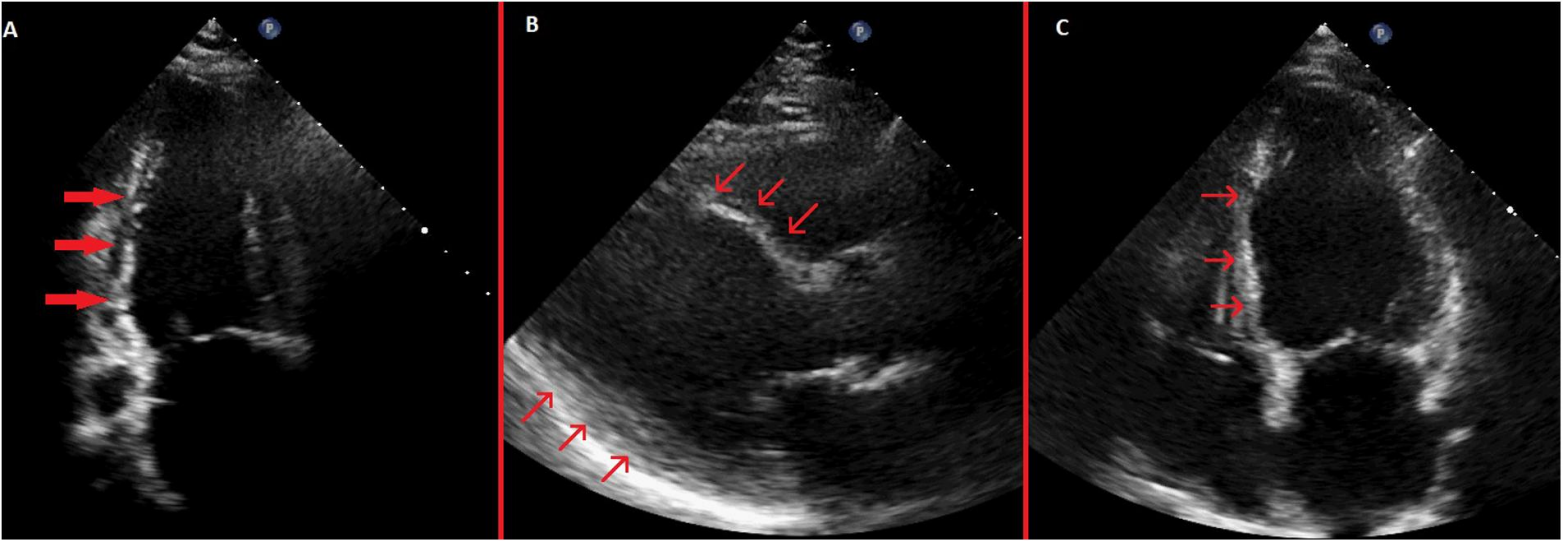
Pre-discharge transthoracic echocardiography images. **(A)** Systolic apical 2-chamber view showing severe hypokinesis of the inferior wall with thinning of the inferior wall myocardium. **(B)** Systolic parasternal long-axis view showing akinetic and thinned anterior septum and inferolateral wall hypokinesis. **(C)** Systolic apical 4-chamber view showing akinetic and thinned basal and mid septum.

Because complete AV block persisted during 5 days of follow-up and LVEF remained severely reduced (below 35%), cardiac resynchronization therapy with defibrillator (CRT-D) implantation was performed using the conventional biventricular pacing approach (Figure 3). The post-implantation ECG showed a biventricular paced rhythm with a QRS duration of 128 ms. Pre-discharge TTE revealed persistent ischemic regional wall motion abnormalities and a mildly improved LVEF of 37% (Figure 4; Supplementary Videos S3–S5). The patient was discharged in stable condition 3 days after CRT-D implantation.

A structured summary of the chronological clinical, electrocardiographic, and echocardiographic findings is provided in Table 1.

**Table 1:** Timeline of key clinical events.

Timepoint	Clinical event	ECG/TTE/Labs
Admission	Typical chest pain; diagnosis as NSTEMI	ECG: Normal sinus rhythm, no conduction abnormality. TTE: LVEF 61%. hs-cTnI: 379 pg/mL.
PCI (Day 0)	IVUS-guided PCI to LAD using jailed-wire technique; major SA became occluded despite wire protection (3-hour procedure).	
Immediately post-PCI (Day 0)	Transferred to CCU; BP 95/62 mmHg (improved with IV fluids)	ECG: New CRBBB (QRS 168 ms), right axis deviation, inferior Q waves, T-wave inversion (inferior + precordial except V2), Qr morphology in V1 and V3. TTE: LVEF: 32%, septal akinesia + inferior/inferolateral severe hypokinesia hs-cTnI exhibited a rising trend
Day 1–2	Hemodynamically stable	ECG: Persistence of immediate postoperative findings TTE: Persistent regional wall motion abnormalities Peak hs-cTnI: 6,451 pg/mL
Day 3 (62 h post-PCI)	Sudden complete AV block (ventricular rate 44 bpm); hypotension and vomiting, prompting temporary pacemaker implantation.	ECG: Persistent CRBBB + right axis deviation (LPFB), complete AV block.
Day 8	CRT-D implantation due to persistent complete AV block + low LVEF	TTE: LVEF 32%
Day 11 (Discharge)	Hemodynamically stable; improved exercise tolerance.	ECG: Biventricular paced rhythm (QRS:128 ms) TTE: LVEF 37%

ECG, electrocardiogram; TTE, transthoracic echocardiography; NSTEMI, non-ST-segment elevation myocardial infarction; LVEF, left ventricular ejection fraction; hs-cTnI; high-sensitivity cardiac troponin I; IVUS, intravascular ultrasound; PCI, percutaneous coronary intervention; SA, septal artery; CCU, coronary care unit; BP, blood pressure; LAD, left anterior descending artery; IV, intravenous; CRBBB, complete right bundle branch block; LPFB, left posterior fascicular block; AV, atrioventricular; CRT-D, cardiac resynchronization therapy with defibrillator.

All LVEF assessments were performed using the biplane Simpson's method.

## Discussion

### ECG findings and conduction consequences

This case describes a clinically important progression of conduction disturbances following occlusion of a major SA supplying collateral flow to a chronically occluded RCA. The baseline ECG showed normal sinus rhythm without conduction abnormalities or ST–T changes. Immediately after SA occlusion, serial ECGs demonstrated abrupt development of a bifascicular block pattern—CRBBB with right axis deviation consistent with left posterior fascicular block (LPFB). This pattern persisted throughout the first three postoperative days, indicating persistent infranodal conduction involvement rather than a transient infarction-related vectorial shift.

At 62 h post-PCI, the patient developed complete AV block, while the CRBBB and right axis deviation remained unchanged. The persistence of a bifascicular block pattern (CRBBB and LPFB) prior to complete AV block strongly supports progressive ischemic injury to the His–Purkinje system. Laboratory parameters, including electrolytes and renal function, were unremarkable, excluding metabolic or reversible causes. Clinically, the appearance of a new bifascicular block pattern in the setting of myocardial infarction is a high-risk conduction profile and warrants prolonged rhythm surveillance and readiness for permanent pacing.

A notable early post-PCI finding was the presence of Qr morphology in leads V₁ and V₃, rather than the classical rSR′ configuration expected in CRBBB. This pattern reflects altered septal depolarization caused by septal infarction, supporting the diagnosis of ischemic injury to the interventricular septum.

Despite the abrupt loss of collateral flow to the chronically occluded RCA, no inferior ST-segment elevation was observed. The preserved inferior wall motion on baseline TTE suggests that the inferior myocardium remained viable under collateral supply before PCI. After the SA was lost, the patient developed acute hypokinesia of the inferior and inferolateral walls, with pathological Q waves and T-wave inversion in the inferior leads. The presence of new Q waves without ST elevation is consistent with non-transmural but clinically meaningful infarction. These findings underscore that loss of a collateral-supplying SA can cause silent yet significant ischemia in territories distal to a CTO.

### Literature context and comparison

Several reports over the past three decades have described late-onset high-grade or complete AV block following SA occlusion during PCI (Supplementary Table S1), most commonly involving the first SA and frequently necessitating permanent pacemaker implantation (2–12). In these reports, conduction disturbances often evolve with temporal delay after the procedure (2–10), although recovery of AV conduction has been documented in a minority of cases, either spontaneously (2, 3) or after successful SA revascularization (5, 10).

Beyond PCI-related mechanisms, non–PCI-associated compromise of SAs due to atherosclerotic stenosis, acute occlusion, or vasospasm has also been reported to precipitate complete AV block (Supplementary Table S2), reinforcing the critical role of septal branches in sustaining His–Purkinje system perfusion (13–16).

Against this background, the present case adds an incremental anatomical–clinical perspective by involving a collateral-supplying major SA responsible for perfusion of a chronically occluded RCA. Unlike previously reported cases that primarily focused on conduction outcomes, our patient exhibited a combined electromechanical presentation characterized by late-onset complete AV block together with marked left ventricular systolic dysfunction affecting the septal, inferior, and inferolateral territories. This observation broadens the clinical perspective on SA occlusion beyond conduction-only complications and highlights the importance of considering both conduction and myocardial consequences when SAs with collateral function are at risk during LAD PCI.

### Mechanistic insights

The main coronary contributors to the cardiac conduction system are the RCA and the LAD. The AV nodal artery most commonly arises from the RCA, which also supplies the His bundle and the proximal left posterior fascicle. SAs arising from the LAD provide the dominant blood supply to the right bundle branch and to the left anterior fascicle, and may also contribute variably to portions of the left posterior fascicle depending on coronary dominance. This anatomical configuration explains why isolated SA occlusion rarely results in complete AV block in most previously reported cases.

SAs display considerable anatomical heterogeneity, and current PCI guidelines do not provide specific recommendations regarding their protection during LAD interventions. However, accumulating case-based evidence—including the present report and the literature summarized in Supplementary Tables 1, 2 suggests that certain SAs may warrant particular attention. First, the first septal perforator emerges repeatedly across published cases as the vessel most frequently implicated in conduction disturbances following occlusion, consistent with its dominant perfusion of the proximal His–Purkinje system. Second, in line with the present observation, SAs that supply collateral flow to territories affected by CTO may have disproportionately greater functional significance; loss of such collateral-supplying SAs can compromise both conduction tissue perfusion and large myocardial regions that had remained viable through collateral support. Third, large-caliber or deeply intramyocardial SAs may be mechanically more vulnerable during PCI and more difficult to rescue if occluded. Taken together, these observations indicate that although routine SA protection is not standard practice, LAD PCI in patients with a dominant, proximal, or collateral-supplying SA may merit a more deliberate protective strategy, informed by pre-procedural angiographic assessment and intravascular imaging.

The mechanisms of side branch ostial occlusion after main vessel PCI include carina shift, plaque shift, ostial dissection, thrombus formation, and vasospasm, with carina and plaque shift being the most common. In our case, intravascular ultrasound (IVUS) demonstrated complete ostial occlusion secondary to plaque and carina shift, without evidence of thrombus or dissection. Despite multiple rewiring attempts and intracoronary nitroglycerin administration, mechanical occlusion persisted.

From a procedural standpoint, management of LAD–SA bifurcations requires recognition of the small caliber, deep intramyocardial course, and limited bailout options of septal branches. In the present case, a jailed-wire strategy was selected based on IVUS findings demonstrating a narrow septal takeoff angle and low ostial plaque burden. The jailed balloon technique—shown to reduce side-branch compromise in conventional epicardial bifurcations (17)—is often impractical in SAs, where incomplete balloon expansion, difficulty in device retrieval, and the risk of stent-edge distortion or entrapment limit its safety. Despite appropriate strategy selection and contemporary rescue attempts, the SA was ultimately lost, illustrating that even optimal protection techniques may fail due to intrinsic anatomical vulnerability rather than procedural error.

Although the SA was lost, one could hypothesize that antegrade recanalization of the RCA CTO before LAD PCI might have preserved collateral flow to dependent myocardium, potentially attenuating the subsequent electromechanical consequences. In this patient presenting with NSTEMI, the close temporal association between loss of a major collateral-supplying SA and the subsequent development of conduction disturbances and new regional wall-motion abnormalities supports a mechanistically plausible relationship rather than definitive causality. Retrospective consideration of alternative revascularization sequencing is therefore speculative and intended solely to highlight the potential vulnerability of collateral-dependent anatomies.

### Therapeutic considerations: CRT-D

Taken together, the persistence of complete AV block requiring pacing support and the development of marked left ventricular systolic dysfunction following SA occlusion prompted consideration of an individualized permanent pacing strategy. Current ESC and AHA/HRS guidelines support consideration of cardiac resynchronization therapy (CRT) with defibrillator capability in patients with ischemic cardiomyopathy, reduced LVEF, and persistent complete AV block requiring permanent pacing after myocardial infarction (18, 19). In this case, CRT-D was preferred over CRT with a pacemaker, as the patient exhibited persistent complete AV block lasting more than five days, an ischemic etiology of conduction disturbance suggesting advanced infranodal conduction involvement, together with a markedly reduced LVEF (32%) associated with an increased arrhythmic risk.

Although the patient met the formal guideline criteria for CRT-D, the timing of implantation in the early post–MI period remains an area of clinical uncertainty. Both the 2021 ESC and 2023 AHA/HRS documents emphasize that early CRT (<40 days post-MI) should generally be deferred because conduction disturbances or left ventricular systolic dysfunction may recover after optimal revascularization and medical therapy (18, 19). Nevertheless, in this patient, complete AV block persisted for over five days despite metabolic stability with new septal and inferior–inferolateral wall-motion abnormalities and severely reduced left ventricular systolic function. Under these conditions, deferring device therapy could have resulted in prolonged bradyarrhythmia, progressive heart failure, or sudden cardiac death. Accordingly, early CRT-D implantation was considered an individualized and pragmatic clinical decision in this specific context.

With respect to device selection and pacing strategy, conventional biventricular CRT was preferred over conduction system pacing (CSP) approaches such as left bundle branch area pacing (LBBAP) or left bundle branch pacing–optimized CRT (LOT-CRT). This choice was primarily influenced by the presence of a broad septal infarction—an area essential for lead fixation and capture stability in CSP techniques. In the acute phase of septal injury, lead placement in necrotic or markedly edematous tissue carries an increased risk of septal perforation, lead instability, and unreliable or fluctuating capture thresholds. Furthermore, the substantial septal fibrosis anticipated during healing could jeopardize long-term CSP performance. By contrast, conventional biventricular CRT provides more predictable lead stability and resynchronization efficacy in this context and avoids additional mechanical manipulation of the infarcted septum during the vulnerable early post-MI period.

Overall, this case illustrates that in rare and highly selected scenarios characterized by persistent complete AV block and severely reduced left ventricular systolic function, early individualized consideration of CRT-D implantation may be clinically appropriate despite general guideline caution.

## Conclusion

This case highlights that occlusion of a major SA—particularly when it provides collateral flow to a chronically occluded RCA—may be associated with significant ischemic injury, a marked decline in LVEF, and progressive conduction disturbances culminating in late-onset complete AV block. Clinicians should recognize that SAs, although often regarded as minor vessels, may be functionally critical for both myocardial perfusion and the His–Purkinje system, depending on the underlying coronary anatomy. Furthermore, this report underscores that even when an anatomically appropriate protection strategy is selected—such as jailed-wire protection in narrow-angle, low-plaque septal bifurcations—intramyocardial SAs may remain inherently vulnerable to carina or plaque shift. Accordingly, SA protection strategies should be individualized based on anatomical characteristics, with careful consideration of the balance between potential benefit and procedural risk in small intramyocardial branches.

The emergence of a new bifascicular block pattern (CRBBB with features of LPFB) after SA compromise may serve as an early marker of evolving conduction system ischemia, warranting prolonged rhythm monitoring and timely assessment for permanent pacing. The delayed development of complete AV block more than 60 h after the procedure further reinforces that conduction disturbances may evolve with significant latency, even in initially stable patients.

Finally, this case illustrates that the combined electromechanical consequences of SA occlusion may support early individualized consideration of CRT-D implantation. In carefully selected scenarios, such an approach may be clinically appropriate despite general guideline caution regarding device implantation in the early post–myocardial infarction period.

## Data Availability

The original contributions presented in the study are included in the article/Supplementary Material, further inquiries can be directed to the corresponding author.

## Supplementary material

Supplementary Table 1 Summary of published cases of jailed septal branch leading to complete AV block.

Supplementary Table 2 Published cases of septal branch stenosis/occlusion (non-PCI) leading to complete AV block.

Supplementary Video S1 Coronary angiogram showing left anterior descending artery culprit lesions and a major septal artery containing collaterals to the right coronary artery

Supplementary Video S2 Coronary angiogram showing loss of the major septal artery despite complete patency of the left anterior descending artery

Supplementary Video S3–S5 Pre-discharge parasternal long axis, apical 2-chamber and apical 4-chamber transthoracic echocardiography.
