# Supplementary material for: Case Report: A collateral-supplying major septal artery occlusion: electromechanical consequences leading to low left ventricular ejection fraction and late-onset complete atrioventricular block
Source: Front Cardiovasc Med. 2026 Feb 13;13:1760781. doi: 10.3389/fcvm.2026.1760781 (PMC12946060; doi:10.3389/fcvm.2026.1760781)
Supplement: Supplementary Table 1 — Summary of published cases of jailed septal branch leading to complete AV block. [file Table1.docx]

Supplementary Table 1. Summary of Published Cases of Jailed Septal Branch Leading to Complete AV Block

| Author (Year) | Septal branch | Collateral inclusion | Baseline conduction defect | Post-procedural conduction pattern | Timing of onset of CHB after septal brachial occlusion* | Outcome | Device therapy |
| --- | --- | --- | --- | --- | --- | --- | --- |
| Furgerson JL (1998) | First septal | No | CLBBB | CLBBB → CHB | Late | Spontaneous recovery of CHB | No |
| Pillai RV (2005) | First septal | No | None | None (direct CHB reported) | Late | Spontaneous recovery of CHB | No |
| Nee LM (2007) | First septal | No | LAFB | LAFB → CHB | Late | Persistent CHB | Yes |
| Kireyev D (2009) | First septal | No | First degree AV block | CRBBB + First degree AV block → CHB (recovered after septal branch recanalization) | Late | Septal recanalization with PCI → CHB recovery | No |
| Sadiq MA (2012) | First septal | No | None | CRBBB + LAFB → CHB | Late | Persistent CHB | Yes |
| Hamatani Y (2014) | First septal | No | CRBBB + First degree AV block | CRBBB + LAFB + First degree AV block → CHB | Late | Persistent CHB | Yes |
| Guragai N (2017) | First septal | No | None | None → CHB | Late | Persistent CHB | Yes |
| Shah GA (2018) | First septal | No | None | None → CHB | Late | Persistent CHB | Yes |
| Nojima Y (2019) | First major septal | No | CRBBB | CRBBB + LAFB → CHB | Late (≈72 h) | Septal recanalization with PCI → CHB recovery | No |
| Didagelos M (2021) | Second large septal | No | CLBBB + First degree AV block | Pacemaker rhythm (native conduction not assessable) + CHB | Immediate | Persistent CHB | Yes |
| Manasrah A (2025) | First septal | No | CLBBB | CLBBB → CHB | Immediate | Persistent CHB | Yes |
| Present case (2025) | First major septal | Yes (collateral-supplying) | No conduction abnormality | CRBBB + LPFB → CHB | Late | Persistent CHB | Yes (CRT-D) |

*Timing categories are defined as follows: “Immediate” = conduction disturbance occurring during PCI; “Early” = within the first 24 hours after PCI; “Late” = between 24 and 72 hours; and “Very late” = occurring more than 72 hours after PCI.

Abbreviations: PCI, percutaneous coronary intervention; AV, atrioventricular; CHB, complete heart block; CLBBB, complete left bundle branch block; LAFB, left anterior fascicular block; CRBBB, complete right bundle branch block; LFPB, left posterior fascicular block; CRT-D, cardiac resynchronization therapy with defibrillator
