# Supplementary material for: Case Report: A collateral-supplying major septal artery occlusion: electromechanical consequences leading to low left ventricular ejection fraction and late-onset complete atrioventricular block
Source: Front Cardiovasc Med. 2026 Feb 13;13:1760781. doi: 10.3389/fcvm.2026.1760781 (PMC12946060; doi:10.3389/fcvm.2026.1760781)
Supplement: Supplementary Table 2 — Published cases of septal branch stenosis/occlusion (non-PCI) leading to complete AV block. [file Table2.docx]

Supplementary Table 2. Published Cases of Septal Branch Stenosis/Occlusion (Non-PCI) Leading to Complete AV Block

| Author (Year) | Septal branch | Etiology of septal artery compromise | Outcome | Device therapy |
| --- | --- | --- | --- | --- |
| Özaydın M (2009) | First septal | Atherosclerotic isolated stenosis | Persistent CHB | Yes |
| Chahine J (2019) | First septal | Atherosclerotic occlusion | Persistent CHB | Yes |
| Bonnet M (2021) | First septal | Atherosclerotic occlusion | Persistent CHB | Yes |
| Takahashi R (2023) | First septal | Vasospasm | Resolved with CCB therapy | No |

Abbreviations: PCI, percutaneous coronary intervention; AV, atrioventricular; CHB, complete heart block; CCB, calcium channel blocker
